# Restructuring education activities for full online learning: findings from a qualitative study with Malaysian nursing students during Covid-19 pandemic

**DOI:** 10.1186/s12909-022-03587-1

**Published:** 2022-07-11

**Authors:** Muhammad Hibatullah Romli, Chan Choong Foong, Wei-Han Hong, Paramesevary Subramaniam, Farahiyah Wan Yunus

**Affiliations:** 1 Department of Rehabilitation Medicine, UPM Teaching Hospital, Faculty of Medicine & Health Sciences, Universiti Putra Malaysia, 43400 Serdang, Selangor Malaysia; 2 Malaysian Research Institute on Ageing (MyAgeing™), Universiti Putra Malaysia, 43400 Serdang, Selangor Malaysia; 3 Medical Education & Research Development Unit (MERDU), Faculty of Medicine, University of Malaya, 50603 Kuala Lumpur, Malaysia; 4 Department of Nursing, Faculty of Medicine & Health Sciences, Universiti Putra Malaysia, 43400 Serdang, Selangor Malaysia; 5 Centre for Rehabilitation and Special Needs Studies, Occupational Therapy Programme, Faculty of Health Sciences, Universiti Kebangsaan Malaysia, 50300 Kuala Lumpur, Malaysia

**Keywords:** e-learning, Emergency, Remote Learning, Health professions education, Qualitative

## Abstract

The COVID-19 pandemic and Movement Control Order have restricted learning activities from traditional face-to-face classrooms attendance shifted to full online learning in the student’s environment. The present study is aimed to explore pertaining issues on full online learning among nursing students and offer a contingency solution. Nursing students from one Malaysian public institution were recruited. The sessions were conducted online via teleconference and were recorded. The data were analysed using thematic analysis with the assistance of QDA Miner Lite software. Twenty-one students participated, resulting in four focus group discussions and three in-depth interviews. Three themes with a total of ten sub-themes were generated: (i) Full online learning has ramifications on life (it is about life; blurred division on education life and personal life; non-conducive environment for learning; health and well-being; human is an adaptable being while the transition takes time), (ii) full online learning is a medium of teaching and learning delivery but with several concerns (the boon and bane of fully online learning; challenges associated with full online learning; coping strategy in handling full online learning), and (iii) Foundation in teaching and learning is the key (role of the educator; teaching and learning approaches; motivation and regulation). A model of practice for full online learning was developed, consisting of some modifications to create a conducive and healthy learning environment. This study embarks on a more structured and standard online learning practice for making the Internet of Things and Industrial Revolution 4.0 concept a contemporary and mainstream education practice.

## Introduction

The sudden abruption of the Novel Coronavirus (COVID-19) global pandemic has disrupted teaching and learning activities. It accelerated the transition from conventional learning to technology-based learning without offering sufficient time and learning space for preparation among higher education institutions, educators, and students. Before the COVID-19, the acceptance of technology-based learning was low, where only blended-learning was accepted in educational practice. In contrast conventional classroom and physical-attendance learning still dominates most of the teaching and learning activities [1]. Full online learning, such as Massive Open Online Course (MOOC) and distance learning, is not popular – although not rejected but does not receive interest – by the mass population [2]. When COVID-19 strikes, this requires drastic changes in every aspect of human life to prevent the plague from spreading. Thus, aggressive action has been taken to maintain distance among persons, wearing face masks and frequent maintenance of good hygiene practices, especially hand hygiene as the vaccine is not yet thoroughly investigated [3, 4]. Governments, including Malaysia, enforced movement restriction orders to prevent and control the spread of the disease [5, 6]. The restriction has directly affected teaching and learning activities where the students are not allowed to attend physical lectures, either quarantined at the hostels or returning home to minimise contact with other people. However, teaching and learning activities need to continue for the students to graduate. Nevertheless, this has brought a dramatic challenge to higher education institutions. Educators need to immediately shift to full online teaching for teaching and learning activities.

Nursing programmes adopt a semester-based system in Malaysia, whereas medical programmes use a block-based system. The semester-based system is a standard system for higher education, including other health sciences programmes. Nursing students have a similar burden in learning theoretical knowledge compared to other health sciences programmes. However, nursing programmes have the highest workload requirement compared to other health sciences programmes; which requires students to complete 56 weeks (approximately 2000 hours) of clinical attachments [7]. Other health sciences programmes (e.g., occupational therapy, physiotherapy, speech therapy, pharmacy, radiology, clinical psychology, environment, and occupational health, biomedical) only require 500 to 1000 hours of clinical/practical attachments.

In addition, nursing programmes requires 60% of its assessments in the form of a final examination [7]; while other health sciences programmes enable 30 to 40% allocation for the final examination, or all assessments can be in the form of continuous assessments [8]. The passing grade for nursing programmes is set to be 50 out of 100 marks [7]; while other health sciences programmes, the passing grade is set at 40 out of 100 marks [9]. This shows that nursing programmes have higher burdens and stress levels due to stringent programme standards.

One major issue for nursing education to take place effectively during the COVID-19 pandemic is because the training relies on precision skills in psychomotor aspects in managing patients, such as performing wound care like cleaning and bandaging. Mastery of psychomotor skills ensures best services and safety are prioritised while reducing errors in performing health interventions. Such training requires close observation and direct experience . Giving and receiving immediate feedback from the educators, supervisors, or preceptors is needed. Compared to other academic programmes such as social sciences or technological sciences, the delivery of health sciences education during the pandemic is majorly impaired by barriers and restrictions of full online learning [10–14].

Nevertheless, higher education, in general, felt the effect of restrictions due to the COVID-19 pandemic. The educators and students faced challenges in negotiating the technology (e.g., learning how to use the tele-conference application), limited resources (e.g., computer, mobile phone), limited facilities and accessibility (e.g., internet connectivity), and limited ability for effective skill development (e.g., hands-on demonstration and practice) [15]. The acceptance of technology use to substitute teaching and learning activities are also reported to be less desirable and creates conflict among the clients (i.e., patients), healthcare providers, educators, and even the public [16, 17]. However, human can adapt to changes and strike to survive, if the person is provided with opportunities to gain experience within the time available [18]. It has become a norm for health professionals education programmes to adopt full online teaching and learning activities. However, limited studies explore the impacts of the COVID-19 pandemic on nursing education. This study aimed to investigate the impacts of full online learning in nursing education from a qualitative perspective based on the experience and perception of the students.

## Method

A qualitative phenomenological design was used to attempt the research aim. The design is appropriate for investigating human experience in its intact setting and making sense of meaningful interpretations of an event that people bring into it [19]. The focus group discussions adopted Ivanoff and Hultberg’s [20] guidelines, while in-depth interviews used Ryan, Coughlan, and Cronin’s [21] framework.

### Setting and Participants

Nursing students at one public institution was conveniently invited to participate in this study. The institution offers a single-intake per year of four-years undergraduate nursing programme with a total of 99 students. The invitation was open to all students which was distributed online via social media, emails, and WhatsApp messages with the assistance of the class representatives (one representative for each year of study). The recruitment was opened for one week, and two reminders were made to increase the participation rate. Students were encouraged to convey the invitation to peers. The participants’ eligibility was for nursing students who have had an active status for the past two semesters and have had online learning experiences during the COVID-19 pandemic. Participants who consented to participate filled up an electronic Google Form (i.e., Expression of Interest form), informing their personal details and indicating preferences (e.g., focus group discussion or individual interview, time to join). The study was conducted between July and August 2021.

### Data collection

Participants were contacted to attend focus group discussions or individual interviews. Each session was conducted via tele-conference application (e.g., Zoom). Communication technology is feasible, convenient, and cost-effective. It offers a less threatening, natural environment and better referral on interview content [21]. At the beginning of the session, the first author informed the participants about the research aim and procedures, and their verbal consent was recorded. The participants were also reminded that the sessions would be recorded. During the session, the participants were allowed to express their ideas in any language preferred (Note: Malaysian students usually use English and Malays languages in a mixture in casual conversations). Casual conversations may contain pidgin language; however, it enables the messages to be robust and meaningful and genuinely captures the participants’ intention and expression in the message; in other words, tacit and cultural understanding in the conservations is preserved [22].

The participants were interviewed based on a list of guided questions (Table 1). These guided questions were developed by referring to previous studies on e-learning [18, 23–26]. The first author developed the questions, and the second and third authors validated the questions for relevancy and clarity [21]. The questions were not used prescriptively, and orders might change. The first author steered the discussion and ensured that optimal information was gathered within the topic of investigation. Spontaneous questions were asked during the session when new or interesting information emerged and required further clarification.

**Table 1:** List of questions to guide the discussion/interview

**Guidance question for the focus group discussion / in-depth interview** • Could you describe your experience with the online learning during this COVID-19 situation with your usual face-to-face teachings? • How do you feel about this new online learning approach? • Do you prefer conventional (face-to-face) learning or the online learning? • How does this online learning differ from the usual traditional leaning? • How does this new learning approach assist your learning process? • What are the advantages of doing this online learning? • What are the disadvantages of doing this online learning? • What are the difficulties you had during this new learning approach in nursing education? • What motivates you to use this approach? Also using it in the future? • How equipped are you in participating in this online learning? • Can you share with us the things that facilitate you in using this new learning approach? • Can you share what teaching or learning approach during the online learning that you like? • Can you share what teaching or learning approach during the online learning that you dislike? • How satisfy you are with the online learning? • Are there any suggestions to improve the delivery of this online learning?	

As the sessions were conducted online, the participants were informed early to be able to sit in a comfortable environment when joining the sessions. Each session consisted of students from the same cohorts (i.e., year of study). For a cohort with more students, the students were equally divided into several groups. The group’s allocation per cohort allows for homogeneity among the group members. The homogeneity facilitates active discussion due to maximum shared values such as learning experience, classmate relationship, peer-equality (absent of junior-senior relationship), and similar overall study experience [20]. The first author acted as a facilitator for the session. An independent note-taker joined the session without participating or interrupting the sessions. The note-taker had no personal association with the participants or the institution. The note-taker was solely responsible for jotting down any critical and interesting points raised in the discussions. The presence of the note-taker was informed to the participants and stayed with their consent. Participants who opted for in-depth interviews, were conducted with the participant and the first author without the presence of the note-taker. No follow-up focus group discussion and interview was conducted.

### Data analysis

This qualitative study’s data management and analysis were guided by Sutton and Austin’s [27] framework which involves content analysis. The audio-recording was listened to several times and transcribed verbatim. To obtain an overall impression for interpretation, listening to the audio-recording, reading the transcript, and referring to the research notes were done simultaneously. The technique called “reading between the lines” was implemented by hearing the participants’ voice tone, emotional expression, connotation, and non-verbal cues to get a feel for the participants’ experience and grasp the underlying message. The analysis was performed using the QDA Miner Lite software (https://provalisresearch.com/products/qualitative-data-analysis- software/freeware/software/freeware/ ). Coding was conducted in the software, and themes were generated by condensing and summarizing codes under a coherent and meaningful message. The research assistant did the transcribing, and the first and fifth authors validated the transcription. The first author performed the coding in collaboration with the second author and then validated by the third and fifth authors. The fourth author then acted as a member-checking process. The summary of the finding was then sent to participants via email for their feedback.

## Results

Twenty-two nursing students participated in the study. In the expression of interest form, most participants comfortable with any methods or prefer focus group discussion, except one from Year 3 and two (out of three) from Year 4 prefer an in-depth interview. After discussions with the prospective participants, the agreement resulted in four focus group discussions and three in-depth interviews. In specific, Year 1 has two focus group discussions (Group A: *n*=5; Group B: *n*=4), Year 2 has one focus group discussion (*n*=5), Year 3 has one focus group discussion (*n*=5), and Year 4 has three in-depth interviews (*n*=3). One participant from Year 1 Group A withdrew at the early stage of the study due being uncomfortable with the topic. The demographic information of the completed participants is shown in Table 2. The time required was 37 minutes for FGD-1, 43 minutes for FGD-2, one-hour-and-six-minutes for FGD-3, and 54 minutes for FGD-4. Meanwhile, the in-depth interview required 21 minutes (IDI-1), 18 minutes (IDI-2), and 20 minutes (IDI-3), respectively. The majority (*n*=19/21, 90%) of the participants briefly responded that they agreed with the summary findings.

**Table 2:** Demographic Information of the Participants (*N*=21)

Characteristic		Frequency (n)	Percentage (%)
Gender	Male	4	19.1
	Female	17	80.9
Ethnicity	Malay	18	85.7
	Others	3	14.3
CGPA	≥ 3.75	3	14.3
	3.50 – 3.74	13	61.9
	3.00 – 3.49	5	23.8
Study year	Year 1	8	38.1
	Year 2	5	23.8
	Year 3	5	23.8
	Year 4	3	14.3
Family income	M40 (RM4,850– RM10,959)	9	42.9
	B40 (<RM4,850)	12	57.1

**Fig. 1:**
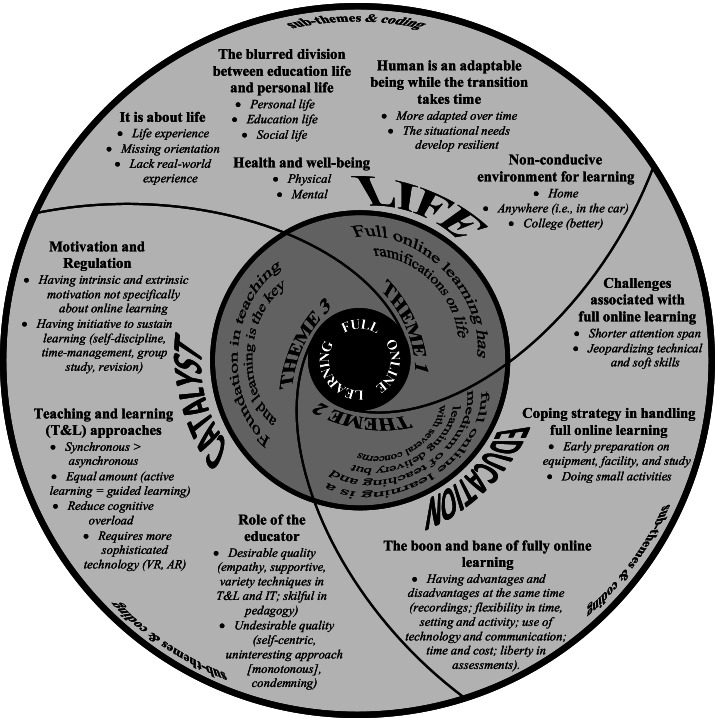
The inter-dependence of themes related to full online learning

Three themes with ten sub-themes were generated, as illustrated in Fig. 1. As the sessions were conducted in pidgin or contained most of the Malay language, quotes were translated into English. Pseudonyms were used to maintain anonymity.

### Theme 1: Full online learning has ramifications on life

Under this theme, full online learning has been explored to impact beyond the education aspect. The effect encroaches into the participant’s personal life and worldview. It can be illustrated into four specific sub-themes.

#### It is about life

Education life is more than just studying. Participants considered student life is not just about attending the education programme and for academic learning only, it is part of gaining life experiences and developmental growth. The participants perceived that full online learning made them lose their university life, especially among the junior cohort. For example, this has been voiced by participants:

“*We were first informed that we can go [physically] to the university and can [physically] register at the university, but suddenly one week before that, it was cancelled. It is a bit of a disappointment as I was excited to experience how the [university] life is… like during the orientation, just online [orientation], so it does not feel like an orientation. It does not feel as it is supposed to be. It feels nothing, just like usual…*” [Participant B, Year 1, FGD 2]

“*Like the seniors during their first year, they went to… like the hospital.. We, the first year, do not have that kind of experience yet. So, it is such a waste during this pandemic for our years.*” [Participant D, Year 1, FGD 1]

Full online learning has retarded their social development and relationship with their peers. This makes trust among their classmates fragile and impacts their learning activity. For example, one participant mentioned:

“*Err… It is difficult to communicate and socialize, ... especially when we are in the first year, just enter [to the university], so we do not know each other, never met, never see each other. Then, when we want to do assignments, it feels a bit of a challenge because we are not familiar with our group partner. So, we do not know the individual’s strength and weakness.*” [Participant A, Year 1, FGD 2]

In contrast, participants in the senior years feel less impacted by social relationships with their peers. They had physically met and built rapport with their classmates before the pandemic struck.

“*…my social life remains the same, nothing changes*” [Participant C, Year 3, FGD 4]

“*…we [friends, juniors-seniors] kept in contact. So, for me, it is less impacted*”. The participants added, “*Maybe for the first year, it is their first year, right, the first intake, the first semester, right? So, they have never been in college, never meet, so perhaps they are not familiar and lack in asking us questions or anything [communicate]… I think that it is…”* [Participant A, Year 4, IDI 1].

#### The blurred division between education life and personal life

Full online learning has eliminated boundaries and a sense of territory in an individual's perception, namely personal role, spatial, and time. During the COVID-19 lockdown, there is a phase where the students need to return to their homes and learn remotely. At the same time, there is another phase where the students are allowed to return to campus but remain only in the hostel while still learning via online. Lack of separation was noted, especially when learning from home.

“*Before this, we know if stay at home, is a place for us to relax, umm… to sleep, all that right. However, now, we need to work from home, everything at home.*" [Participant B, Year 3, FGD 4]

“*Because when we are online… we… umm… we are also a child at home, so we need to help do the house chores, all the other things. So, we need to divide our tasks more.*” [Participant A, Year 1, FGD 1]

#### Non-conducive environment for learning

Online learning creates a non-conducive environment compared to traditional brick-and-mortar class attendance for teaching and learning either for the students or the educator as notified by the participants. There are many challenges and disturbances available. A proper environment such as a college or dorm is more conducive for learning, contributing to feel as a student than at home.

“*…when we at college, we have the surrounding that facilitates us as a student. So, when I am at home, the environment is different; as a daughter, as a sister, we have many responsibilities.*" [Participant A, Year 4, IDI 1]

“*…sometimes, I [attend] the class in the car. It is one of the disturbances.*" [Participant C, Year 3, FGD 4]

“*…we do not have the equipment at home. Like physical learning, we go to the university; there is a facility, everything is there, that is one thing. Like, okay, for example, if we use of what we have at home, the feeling is different.*” [Participant C, Year 2, FGD 3]

“*…the lecturer is also at home. So, they also do not have the equipment to show us, to demonstrate to us. So, they use many videos to show during the class. However, that is still constricted.*” [Participant B, Year 2, FGD 3]

“…*the equipment that we need to use, we do not have it, and we cannot practice by ourselves at home. If we want to buy it, it is expensive. So, what we learned is just theory, like the theory of how to take blood pressure. Just a theory as we cannot do the hands-on practice. So, it is difficult. Just theory without skills.*” [Participant A, Year 1, FGD 1]

#### Health and well being

Most participants voiced that they feel exhausted with online learning. Some of the participants felt stressed and bored with the current condition. The exhaustion is manifested into a physical status where the participants feel a lack of energy, tired and rapid energy drained by attending the online learning all day. Online learning makes the participants lose physical activity opportunities and increases their sedentary lifestyle, even when compared with physical learning:

“*[in physical learning] always moving around, like going to the class and move to another class, Umm… but for me, the online learning is more tiring because we just stay at one place and focus on the computer, and then I feel like our eyes sore.*” [Participant C, Year 1, FGD 2]

There are also complaints of physical health issues among the participants, such as vision problems and headaches:

“*…before the [full] online class, my eye problem is not so bad. However, now, my eye is more than 100 [myopia reading] where before this it was just 50 [myopia reading] like that…*” [Participant C, Year 3, FGD 4]

“*Like me, because I have migraine, because, err… always look at the computer. It makes me have a headache, like many days I have been having a migraine, like that…*” [Participant B, Year 3, FGD 4]

However, a participant considers that full online learning is a reasonable and proactive effort to protect them from the exposure risk of the COVID-19 pandemic.

“*…as we know, right now is the [COVID-19] pandemic. So… when we do online learning, at the same time we can take care of ourselves, our health [from exposed to the risk of COVID-19 infection].*" [Participant A, Year 4, IDI 1]

#### Human is an adaptable being while the transition takes time

The participants prefer traditional physical learning more than full online learning. However the participants is becoming more open and receptive to the notion of online learning as part of standard mainstream practice. They can continue with the online learning if needed. Not only students but educators are more adaptable to online learning after the period.

“*…because there is [almost] two years with online learning. So, quite okay. Nothing weird. Umm… for me, everything is okay now. Like the lecturer also can adapt.*” [Participant B, Year 4, IDI 2]

The participants prefer for some classes to be online such as those related to theory learning (i.e., cognitive) while requesting for physical learning for any topic that requires skill training – not only the practical (i.e., psychomotor) but also soft skills (i.e., affective).

“*…if for the online, like theory learning is okay. If face-to-face [physical] maybe something that we need to demonstrate… umm… procedure that we need to do.*” [Participant C, Year 4, IDI 3]

### Theme 2: Full online learning is a medium of teaching and learning delivery but with several concerns

The participants consider online learning as an alternative for delivering a lecture and learning activities; online learning is not a unique learning technique or approach. Therefore, many learning approaches in physical learning are transferable to online learning with some modifications. However, online learning partially brings the values and meaningful experience reflected in physical learning. The participants perceived that physical and online learning has its strength and limitation.

#### The boon and bane of fully online learning

A fine line that divides between the advantages and disadvantages of online learning. Several aspects were identified, such as flexibility in time, setting and activity, communication aspect, availability of back-up material (i.e., recording), financial concern, and participation in assessment.

The participants reported that full online learning had provided them and the educator with flexibility in time, setting, and activity. The flexibility provides an opportunity for the participants to be independent. The class can no longer be made during the day or hassle to reschedule due to lecturer unavailability (such as finding an empty slot or booking the facility). However, it can conveniently be made available at any time, even at night. It also saves time for the participants where minimal effort requires self-care and travelling. Moreover, online learning makes them able to attend the class whenever possible. In addition, the students can do other activities while attending the lecture, such as having a meal or doing house chores.

“*[The] good point is, we do not have to be there. We can even [attend the class] in the car. I saw some of my friends do that.*” [Participant C, Year 2, FGD 3]

However, the flexibility given too much freedom has created uncertainty in scheduling and interrupting participants’ time. The participants may compromise on class attendance, such as near-missed incidents of non-attendance. The facility issues such as internet connection may require them to find a place such as a kitchen or uncomfortable corner of the house . There is also a misperception among some educators or family members who underestimate students' burden and availability.

“*However, in the flexibility, sometimes it is too flexible until it disturbs our rest time… I have experience attending the exam at night.*" [Participant C, Year 2, FGD 3]

Communication has become more feasible where the participants can communicate with their classmates using teleconference medium without requiring a place to meet. Communication can be done through various telecommunication methods such as WhatsApp or Telegram. This is not limited to their peers but also to the educators, whom they can email any queries they have rather than immediately meet the educator after the class finish when in traditional physical learning.

“*Online for me is easier to meet. Even though we cannot do work physically, the communication is easier, like we want to interact just via Google Meet*” [Participant B, Year 3, FGD 4]

“*…but then on the online learning as we can only rely on the WhatsApp, or maybe a google docs, something like that to share information.*” [Participant D, Year 1, FGD 2]

However, communication using technology has limitations where delay in response is tangible, feels a bit difficult in manoeuvring the technology (e.g., extended typing), missing the intangible message (i.e., non-verbal communication), and lack of authentic relationship (communicated for the sake of work and facts, rather than social mingling). Some participants prefer the conventional communication style of a face-to-face meeting.

"*Right now, we are in the online class, so if we want to ask or chat with our friends, we need to WhatsApp. I am quite lazy to type to… umm… ask my friends. I prefer to meet and directly ask, easier because what we want to know can get it on the spot. So if through WhatsApp, we need to wait for the person. Perhaps the person is doing something else whatsoever, so, after a long wait, like what we want to ask has… is no longer interested.*" [Participant B, Year 2, FGD 3]

Most of the participants feel that the availability of the class recording is beneficial for them to refer for any missed information and revision. They can return to the recording at their convenience. The recording can be a backup if an unwanted event occurs (i.e., lost internet connection, parents calling, doing chores). However, this also creates a latent attitude among the participants where they do not give full attention during the class as they know they can refer to the recordings. Some students admit that hearing the recording is a bit dull and makes them sleepy.

“*[D]uring the study week, I will watch back the lecture recordings, you know… Umm, sometimes I think if face-to-face, I do not have a recording. Like me, if I do not understand, I need to meet the lecturer back. However, with the recording [available], I can watch back and understand what I learned previously.*” [Participant B, Year 1, FGD 2]

“*…Frankly speaking, I feel sleepy watching the recording; I rarely watched it… However, yeah, the recording is also valuable. For example, I can review back lectures of last semesters on statistics and now understand it better for my research project*” [Participant B, Year 4, IDI 2]

Some participants perceived that online learning made them save more money and reduce cost-related expenses such as preparing the assignment materials. However, other costs may increase, such as the subscription to internet service and those students who do not have the proper equipment for learning need to find a way to get the equipment such as laptops. Having proper information technology equipment (i.e., computer, laptop, smartphone) and facility (i.e., mobile data, Wi-Fi and internet connection) is crucial to ensure comfortable and successful online learning.

“*…for me, online learning also has its advantages. Like, save time, safe energy… oh yes, save money.*" [Participant C, Year 1, FGD 1]

“*At first, I need to share a laptop with my brother, because… err… I do not have my own laptop at that time. So, it is quite difficult; for example, I have a class that clashes with my brother's class. Then, I knew that Zoom could be installed on the phone. So, I just use the phone afterward. Hmm…*” [Participant B, Year 1, FGD 1]

“*…students do not have much money. So, difficult to pay… like buying the internet plan…*” [Participant D, Year 1, FGD 1]

The students presume that online learning has created liberty in their assessment and makes them more prepared to achieve a better grade. However, some participants consider this has increased the burden on preparing the assignment. In addition, assessment, especially examination conducted online, creates conflict and queries the students’ integrity and ethics when approaching the assessments.

“*…and, even sometimes, we… like… Umm… we prepare the script earlier. So, it is easy for our presentation.*” [Participant A, Year 2, FGD 3]

“*…mostly… [the assignments] need to do video. So, personally, it is one of the biggest constraints for me to do the assignment. It feels like… to prepare, okay, whom we want to make patient, and then the equipment, where to do it. And then, it is not just about shooting the video only. It's like we need to watch back; if not okay… then we need to re-shoot.*” [Participant B, Year 2, FGD 3]

“*Final exam right, umm… each subject… each course, like, different. Like ours, we need to answer at that time and open the camera [for proctoring]. So, there are other courses I saw, like the lecturer gave them the whole day and it is up to them, they can discuss with friends, seek answers, etc. Umm… so, it is sort of unfair. It is not standardized.*” [Participant A, Year 3, FGD 4]

However, the participants consider that all these depend on the individuals to decide how they utilize online learning to scaffold its benefits or drown into its affliction.

#### Challenges associated with full online learning

However, online learning identified several challenges, such as a shorter attention span. The participants deemed that they were easily distracted and jeopardized their technical skills.

“*[F]rom what I observe, in an online class, I can focus only 20 to 30 minutes, and after that, even I try to focus, it will be challenging. When the lecturer says [the lecture], I just see and hear but cannot understand what the lecturer says. If compared to a physical class, I can maintain my focus for one to one-and-a-half hours… like that.*” [Participant C, Year 3, FGD 4]

One significant barrier to full online learning is that it only caters mainly to cognitive purposes, while limited for affective and negligible for developing psychomotor skills . The participants affirmed that the nursing course is technical and requires skill development. However, the full online learning is inadequate to train their nursing skills, especially the new skills. In addition, there are contradictions found between the procedure taught as it uses commercially and publicly available video, which may not be similar or inapplicable in actual local clinical practice. Thus, the participants feel they need to re-learn, which has taken more of their time.

“*Like before, , we practice in the nursing lab after the theory. Nevertheless, [in online learning] when we finish with the theory, it just ends. It is difficult for us to understand things when watching a video because it is a skill, right. It cannot be like that; when we learn a theory, we can memorize all that; however, when it is a skill, yes, we can memorize, but it is gone when we want to do it. Because it is experiencing, based on experience.*” [Participant B, Year 3, FGD 4]

Not only that, the participants perceived that their obtained skills were also deteriorating. This is because they have limited opportunities to practice the skills again.

“*…one more, our existing skills are also diminishing. For example, we are now at the senior year, so when we are not going towards, or going to the nursing lab for practice whatever we learned and have done, we like… not remember it [skills]*” [Participant E, Year 2, FGD 3]

#### Coping strategy in handling full online learning

The participants reported several strategies to stay alert and sustain their online learning participation and independent learning.

“*for online [learning], make sure, if do not have Wi-Fi, then buy the internet data, all that to smoothly join the class, as worries being disturbed due to connection problem. Like if do not have room (for study), try to find a place like in the kitchen, far from others, to focus on the class.*” [Participant B, Year 3, FGD 4]

For staying alert and sustain their participation in fully online learning, especially the lecture, the participants implement small interval activities for a quick divert and refresh. The participants may move around, do other activities, or have a small meal when they feel tired or uncomfortable.

“*…like before the class, I will prepare snacks or something to eat… Umm… during the class. So, I can focus because it is difficult when hungry.*” [Participant D, Year 3, FGD 4]

"*For me, I will play with the handphone, or otherwise, I lie down, go to the toilet, have a drink, walk a bit, then sit back in front of the computer.*" [Participant D, Year 1, FGD 1]

For their independent study, the participants modified the usual learning approach they had done before online such as group discussion that was previously conducted by meeting outside and gathering in a group physically into meeting virtually via teleconference application.

### Theme 3: Foundation in teaching and learning is the key

Techniques or strategies in teaching and learning play a pivotal element to ensure the continuity and sustainability of learning. The techniques are general, and not limited to only technology but emphasize educational theories, individual approaches and the conventional concept of teaching.

#### Role of the educator

In participants’ opinions, educators need to excite the teaching by avoiding being monotonous in the lecture, not just reading the slide, and applying various activities to make the learning session interactive and engaging.

“…like some of the lecturers, ermm... that just read the slides, as I said before, and then did not give any explanation. Maybe s/he include all the… all-important points in the slide. We can say that, and then each part s/he just read-only. *laughing*. ermm... I think, ermm... this one also let us feel bored. We do not want to just listen, and go through the slide. Because aaa... if the lecturer just read the slide, we can also read [it] by ourselves.” [Participant A, Year 1, FGD 2]

“*…I think that s/he teaches with enthusiasm. Before s/he teaches something, s/he will relate the thing with… current… general issue. So, it makes me like, ‘Wow!’ so interesting on how s/he teaches. S/he likes so eager to make the students understand on what s/he tries to deliver.*” [Participant B, Year 1, FGD 2]

The participants mentioned that they less appreciate total independent learning, such as replacing the lecture with activity and presentation without validating the information presented. As they feel uncertain about the information they gain and less confident about what they learn, the participants require confirmation from the educator.

“*[A lecturer] likes to replace, right. Sometimes, s/he just replaces the class with lots of assignments.*” [Participant E, Year 3, FGD 4]

In addition, participants appreciate educators who are empathetic and not condemning. The participants appreciate educators who are enthusiastic about teaching and if the procedure is carried out by the educator rather than showing a video from the internet.

“*…more empathetic. Like, sometimes a student says coming a bit late due to an internet problem, please do not respond like "Oh, you can prepare early". The internet, sometimes is beyond our control. Hmm… I found lots of lecturers like that. I feel like they lack empathy. It actually will affect the student's emotion.*” [Participant C, Year 2, FGD 3]

"*…some likes condemning, like, err… s/he said, "I have told you to study by yourself, right? I have given a task for you to do… to look at" like that. Furthermore, like mad at the students because we cannot answer his/her question. It is not because we do not want to answer the question (correctly), but we do not understand.*" [Participant A, Year 1, FGD 1]

"*Maybe lecturer or the department can do… or request the clinical instructor or anybody to… err… record a procedure video for us to watch. Because when we watched the procedure video on YouTube… err… yes, it can add our knowledge, but the procedure is clashed (not suitable) with the standard checklist we have.*" [Participant D, Year 2, FGD 3]

The participants believe that the educators can improve themselves by having training related to technology use and technology-based learning applications.

“*Both (students and educators) need… urm… training or courses to improve online learning and teaching skills. Because not all lecturers know how to use this online [learning], even though they have been for a year like this. There are many things to explore, a lot… material, apps, websites that can be used for teaching purposes to make our learning more interesting. Moreover, for the students, we would like to improve our note-taking skills.*” [Participant C, Year 3, FGD 4]

#### Teaching and learning approaches

Although the participants believe that asynchronous learning, such as lecture recording, is beneficial for them, the participants more appreciate synchronous than asynchronous learning. The participants feel synchronous learning is more meaningful and has a 'soul' of teaching and learning than asynchronous, which feels 'disconnected'.

"*Although the online class has recorded lectures, respect for the lecturer is important for me. So, I still attend the online (synchronous) class. I feel it is better to attend the class than not coming and just watching the recording.*" [Participant D, Year 2, FGD 3]

The participants seemed to appreciate activities while still expecting guided learning to validate the acquired knowledge. The participants identified that a non-marking quiz is beneficial to help them immediately revise, focus on the lecture to prepare for the quiz, and understand what is important in the lectured topic. The participants request all-participate quiz activities such as using game applications; the participants are uncomfortable with individual-calling question-answer activity.

“*…some lecturers have done… Umm… Q&A (quiz) session but not directly to a particular student, but instead s/he used Kahoot*^*TM*^ *(quiz game application), so all students can join. Sometimes some students are shy to answer (direct asking), but with Kahoot*^*TM*^*, everybody participates, knows right and wrong (answer) simultaneously.*” [Participant D, Year 3, FGD 4]

A simple group activity in the middle of the class is another approach highlighted by the participants. The group activity is perceived as helping break the dull pattern in online learning while making them more active. However, the educator needs to be wise in allocating ample time, not a complex task, conducive and non-threatening environment, and a supportive attitude with immediate constructive feedback.

"*…like a way of one lecturer to maintain us energetic until the end of the class. Where s/he always gives us a small task, group work just around 10–20 minutes for a short presentation. So, we feel refreshed and focused.*" [Participant D, Year 2, FGD 3]

Excessive information in lectures makes the participants feel overwhelmed and unable to absorb the information thoroughly. The participants hope that the educator may point out which information is critical. A gap between each topic is required to help the students relax and rehearse the information. The participants believe that various teaching approaches implemented during online learning are helpful for them to stay alert. However, the implementation should be nice, not too crowded and, confusing. This includes using uncrowded PowerPoint slides, interactive slide preparation, video, simple reading materials, and demonstration.

"*the lecturer cannot like include everything into the slides…*" [Participant D, Year 1, FGD 2]

"*I like with a technique that is not straight lecture, like if the class is two hours, then that two hours is a lecture. I prefer, some of the lecturers that lecture like one hour, then have gaps to watch the video, do discussions, gave questions, and asking for our opinions about the video.*" [Participant B, Year 3, FGD 4]

Skills learning are the most impaired due to fully online learning. The students hope that the lecturer can replace the skill teaching using other methods. Utilizing sophisticated technology such as virtual simulation can be explored, which provides richer haptic feedback.

“*It is like this; it is like the Labster (i.e., virtual lab online webpage), make a simulation program. So, like, they make you feel like you are in the lab like you can pick up the test tubes and insert all the kinds of solutions. It is a simulation program, so like you have the interactive lab. It is like, you feel like you're in the lab. So compared to that, I think that's more interactive comparing to like just watching a video, a practical video it's like watching somebody do like all those things, like compared to that I am more like to have an interactive simulations program, for me to try things out instead of just watching videos, yeah.*” [Participant D, Year 1, FGD 2]

The participants appreciate prepared educators, such as providing the lecture slide early, the assignments early, and informing on any schedule change early. The participants find it difficult if the schedule, either class or test made abruptly or unexpectedly.

“*The lecturer does not give the notes early. So, in consequence, we are rushing to jot down the notes. If the lecturer gives us the notes early, we can refer to it and just note down any important points during the lecture.*” [Participant E, Year 1, FGD 1]

“*Pressure from the lecturers who like… umm… like to give lots of assignments, sometimes as they wish, change the class at the time they want, and suddenly have a test.*" [Participant C, Year 3, FGD 4]

#### Motivation and regulation

Some participants had no issue with online learning, especially those in their most senior year. However, several participants feel stressed, and others are demotivated with full online learning. The participants continue with the online learning is not because of the online learning itself but driven by other factors such as their willingness to learn, thinking about the future, wanting to deliver the best for patients, finishing the study, maintaining academic achievement, achieving ambition, family, friends support, financial, and respecting the educator.

“*…what drives me is my family and my future.*" [Participant A, Year 3, FGD 4]

“*…I just want to pay my [study] loan after I finish my study. I have studied right.*” [Participant E, Year 3, FGD 4]

“*…I do not have any motivation for this online learning. However, I have to learn online because I do not want to fail...*" [Participant C, Year 1, FGD 2]

“*…my motivation is from our… my surroundings and my friends because all my friends also study online and then, ermm... So, I want to follow them, I don't want to isolate myself.*” [Participant A, Year 1, FGD 2]

The participants implement several approaches to self-regulate their learning. Some participants do early preparation before attending the online class. Other participants consider group discussion as beneficial for helping in their learning. Some other participants have the initiative to contact the educator for further learning and clarification.

“*…we cannot procrastinate because if we do, then the assignments will be piled up. So, every day we attend the class, we [also] need to study the subject of the day immediately, we cannot just leave like that. Like, if something we do not understand, we cannot delay, then near to the exam will study back. No, I cannot. We need to make notes immediately. Hmm… Because, if we wait until the final exam… Uh, study week to start studying, we cannot, because too many subjects. Furthermore, the time during the study week is not enough.*” [Participant A, Year 1, FGD 1]

“*I will study early, quite early. Like after the class, I will re-watch the recording or revise my note. Hmm… If I do not understand anything, I will… message the lecturer.*" [Participant E, Year 1, FGD 1]

“*We did group discussions on a certain topic, like who do not understand [can] learn together.*" [Participant B, Year 2, FGD 3]

Most of the participants believe that it is up to one's initiative and individual responsibility to ensure successful participation in learning. Self-determination, such as self-care, preparing the equipment for learning early, ensuring a good internet connection, and preparing an environment conducive to learning, helps to make their learning smoother and hassle-free. In addition, the participants consider time management is an essential skill for them to go through online learning. The participants hope there is also training available for them in time management related to online learning, such as note-taking skills and assignment preparation, and training available on team working in online learning.

“*…for me, it [online learning] disciplining myself, and teach me to appreciate the time.*" [Participant A, Year 4, IDI 1]

“*…we need to have… umm… a training on time management, for students. And then… one more is about teamwork.*” [Participant A, Year 4, IDI 1]

**Fig. 2:**
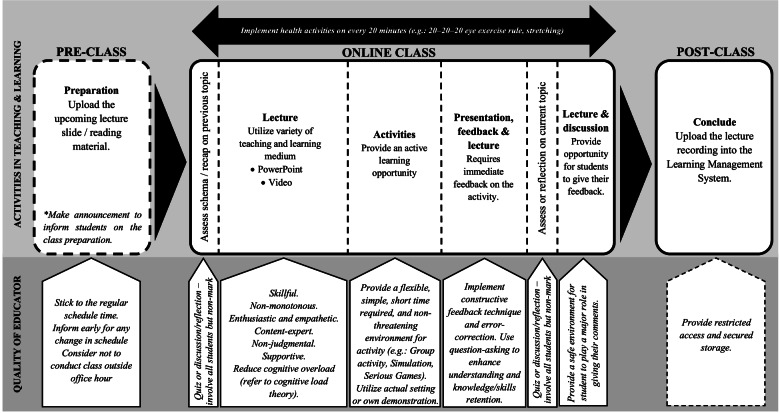
Practice framework developed from the qualitative findings

The findings reported above were synthesized to develop a framework guiding educators to escalate a better education practice. The practice framework integrates learning approaches and educator qualities for a single lecture or class activity. The practice framework is shown in Fig. 2. The practice framework is a systematic organization of learning activities to ensure an exciting learning environment with active engagement of the students in the learning process and good educator participation and preparation for teaching. The practice framework considers for health and well-being of the learners, and healthy behaviour is also incorporated. However, the framework is flexible, especially on the activities during the lecture, where it should not be followed linear or prescriptively.

## Discussion

Online learning is an alternative medium for teaching and learning activities during the COVID-19 pandemic. Online learning transfers teaching and learning approaches from a traditional physical brick-and-mortar environment into a digital platform [28]. The success of online learning is similar to traditional physical learning; it emphasizes active participation, educators’ character, and application of various types of learning activities such as gamification, student-centred learning (i.e., problem-based learning), small group learning (e.g., team-based learning), and multimedia use in didactic lecture (video, interactive and simple PowerPoint presentation). These elements were found in the current study and supported by the literature. For example, a meta-analysis indicates that online multimedia learning is more effective than didactic online lectures [29]. A systematic review found eight elements can become enablers or barriers to e-learning [30]. The eight elements are: (i) facilitate learning; (ii) learning in practice; (iii) systematic approach to learning; (iv) integration of e-learning into curricula; (v) poor motivation and expectation; (vi) resource-intensive; (vii) not suitable for all disciplines or contents, and (viii) lack of IT skills. This current study resembles elements from this systematic review. The use of multimedia and various learning techniques facilitates learning rather than just relying on didactic lectures throughout the online class.

Attention should be given to the information provided during online learning. Online learning utilizes multimedia approaches such as PowerPoint presentations, video, pictures, and audio-visual equipment. This has given rich sensory input and a high cognitive processing burden. The extensive use of cognitive processing creates a shorter attention span due to fatigue. This is agreed with previous literature where online learning created work and information overload received from instructors [15]. Mayer’s cognitive theory on multimedia learning [31] mentioned that the brain does not interpret the inputs in a mutually exclusive fashion but emphasizes the finite ability of cognition to process the information and make sense of the information by creating an active mental representation. The cognitive process needs to filter, select, organize and integrate information based on prior knowledge. These rely on the three memory stores: (i) sensory memory, (ii) short-term and working memory, and (iii) long-term memory. Providing too much information overcrowding the sensory input and left small room for the learners to absorb and understand. Therefore, the educators need to be aware of overcrowding the teaching and learning activities to reduce the cognitive load for successful learning.

In this study, the learners believe that even with the constraints, they will become good and competent practitioners based on their initiative and will. The underlying knowledge they gained from the course, will prepare them to retrieve such information when they encounter the issue in practice. Therefore, what is more important for educators is strengthening the foundation and concept of existing teaching and learning rather than creating and exploring new theories. The current study found that educators have limited knowledge and pedagogical concepts. It was found that some educators rely on didactic lectures, slide reading, and a teacher-centric approach. Unlike school, educators in higher education institutions are not compulsory to have education qualification [32]. However, having formal training and qualification in education was found to improve the educators pedagogical and education related competency and learning experience for the learners [33, 34]. Pedagogy stresses the requirement of class attendance, guidance, and frequent contact with peers and teachers while nurturing critical thinking and active participation in the learning process [35]. Encouraging independent learning is desirable. However, undergraduate students still require guidance as they are inexperienced and require prompt validation of their knowledge [25, 36–39]. The educators should adapt and equip with pedagogical skills for online learning and have a positive quality of supportive educators. One crucial skill for educators is small group learning and management [40]. Group activities promote enquiries through interaction and active participations and breaking the dull atmosphere [40]. Participants in this current study also appreciate a prompt and simple group tasks to make the learning environment vibrant. For the educator’s quality, it is essential for the educator to be critical in content knowledge and reasoning, such as providing a real-world example, have good intrinsic and personal attributes, efficient teaching skills, having positive relationships with students and a supportive learning environment, having good communication skill, the clinical teacher is the enthusiasm with the work, provision of skilful feedback, fostering collaborative learning, understanding expectations, and organization and planning [41, 42]. The educators themselves are the teaching and learning medium; the attitude, behaviour, and gesture can attract or distance the students from learning.

Adapting to a transition in a life event could be a stressful situation. The adaptation requires time for an individual to adjust to the new norm. The transition is an inevitable process that needs to be overcome by learners or educators. The stressful situation is unavoidable, but the adaption can be expedited and tolerated. The current study’s findings are contemplating the criteria for dealing with such a transition [18]. This current study shows that both learners and educators are struggling in conducting online learning at the beginning of the pandemic but becoming more competent after a period of time due to the opportunities to learn while practically teaching the online class. Another study supports this, showing that learners and educators are gradually receptive to online learning over time [43]. A conducive environment for learning, such as a dedicated area at home or being at the college (with internet accessibility), enable students to feel relieved, secure, and mentally prepared to learn. It is undeniable that the booming of technology has created many resources beyond the capability of learners and educators to explore. Some lack IT skills; however, learners and educators must identify and select essential technology that can optimally help for improve knowledge acquisition and skills development. This is in line with the literature, which mentioned that the early use of online learning due to the COVID-19 pandemic had exposed the learner to inadaptability and unfamiliarity with the new online learning environment [15]. At the same time, after a period, the situation is more accepted and adapted [43]. Stress is the culprit for learners to participate in online learning successfully.

In this current study, some students felt mentally and physically exhausted. There are impacts of COVID-19 online learning on mental health [15]. However, it might be contributed by movement restrictions and fear of infection, also known as the quarantine effect. A systematic review and meta-analysis found that quarantine impacts are significantly higher than non-quarantine samples on levels of depression, anxiety and stress [44]. This can be noticed from the current study where the students mentioned their stressful life as they had to alter their daily routines. Online learning is merely one of the life events they need to adapt to. Therefore, this current study recommends that learners divide their personal and study matters. Morale support is essential, and having supportive family members and friends is the social pillar for the learners. Coping strategies in surviving online learning can be successfully extracted from motivation and regulation.

The current study is parallel with a past study reporting that online learning due to the COVID-19 can be demotivating [45]. However, several motivations were explored and possible exploited to sustain participation in online learning. According to self-determination theory, there are three types of motivation: (i) amotivation, (ii) extrinsic (external regulation, introjected regulation, identified regulation, integrated regulation), and (iii) intrinsic motivation [46]. The amotivation of the learners on online learning exists, such as wanting to give up the study. However, in this current study, the extrinsic and intrinsic motivations were found to drive them to continue their studies, such as thinking about their family members, finishing their studies, maintaining good academic results, thinking of providing exemplary service to the patients, and thinking about the future for work and paying loan.

This current study is parallel with the previous studies, which identify self-regulation as the key for the learners to maintain focus in online learning [15, 43, 47]. Self-regulation facilitates students to accomplish a task better by providing opportunities to reflect on their learning [48]. The learners critically evaluate their learning and living styles during the pandemic, reflect on the online learning experiences, and generate contingency plans if necessary. For example, the recording (i.e., playback) is considered an important medium that helps students revise. Self-discipline and time management are additional strategies for them to self-regulating for online learning. The learners also implement alternative study methods, study before the online classes, and optimally utilize telecommunication technology such as emails, messaging apps (e.g., WhatsApp, Telegram), and the internet (i.e., a website search, Google Meet, Zoom) with the peers and lecturers. Self-regulated quickly switched one strategy to another, such as contacting peers or self-searching the internet, after encountering an unsuccessful strategy (e.g., delayed response from an educator).

One vital limitation of online learning is on developing technical and psychomotor skills. This limitation was consistently highlighted in the literature that explored stakeholders’ perceptions of [15, 49–52]. However, with the advancement of technology, educators could explore possible technologies in helping the students to develop psychomotor skills amidst the pandemic. For example, virtual and augmented reality are potential to be used in teaching technical skills. Virtual reality is a technology that is a “computer-generated reality, which allows learners to experience various auditory and visual stimuli experienced through specialized ear and eyewear, such as head-mounted- displays” [53 p.2]. Virtual reality has been developed in nursing education, to cater to complex tasks and teach procedural skills, soft skills (i.e., confidence, communication), and psychomotor skills [53]. In a systematic review, virtual reality was found plausible to improve knowledge acquisition, increase self-confidence, efficacy, and satisfaction levels, and reduce anxiety [54].

Meanwhile, augmented reality “allows for digitally generated three-dimensional representations to be integrated with real environmental stimuli” [55 p.1]. Students are receptive and have a higher interest in using augmented reality, providing a safe and error-negotiating learning environment [55]. Augmented reality is perceived as helpful in enhancing knowledge and understanding, technical and social skills [55]. Virtual and augmented reality are also plausible for clinical assessments [56], which will be meaningful to provide various assessment experiences than the currently available, and cater to psychomotor assessments. The use of sophisticated technology is expected to become mainstream in the near future for health professions education. Nurse educators should prepare for technological practices [57–59]. The technologies are contemporarily investigated in Southeast Asia practice and have positive reception [15]. At the institution where the current study was conducted, virtual and augmented reality is yet to be practiced in teaching and learning. These technologies are emerging, show potential, and are increasingly gaining attention; however, future investigation is required on its effectiveness including physical feedback such as haptic sensors and responses.

### Strengths and limitations

Trustworthiness is the strength of this study. Several steps and measures were taken to ensure the trustworthiness of the findings [60]. For credibility, most of the participants responded and agreed with the summary of findings. Member checking may improve the credibility (i.e., validity) of the findings. Meanwhile, rich data contributes toward a thick description that may comprehensively cover the nursing students’ view on full online learning [61–63]. The present study does not utilize the conventional data saturation but utilize the data richness method to reach a *thick description; “refers to rich, thorough descriptive information about the research setting, study participants, and observed transactions and processes.*” [63; p.1453]. Although saturation is not conducted, however, the recurrent pattern that emerged in each session indicates the shared perception among the participants.

Furthermore, the number of participants was approximately 20% of the nursing student population in the institution is substantial. Many literatures suggested purposive sampling for qualitative study [62]. However, convenient sampling provides a higher opportunity for interested individuals to participate in the study and may result in a bigger sample size and broader representation [61]. Then, convenient sampling recruited individuals voluntarily who were interested in the study topic; thus, the participants may have been more prepared, eager to share their ideas, and deeply engaged in the session. There is no consensus on participants’ size to achieve an optimal data analysis. However, this present study is expected have achieved the desired size. Optimally, a qualitative study requires around 4 to 18 samples and is divided into three focus groups to achieve data enrichment [61, 62] – where it is suggested between 4 to 8 members per group [20]. The first author conducted a Zoom session with two note-takers and discussed the findings for confirmability. Note-takers proposed a minor modification to ensure a more transparent and accurate interpretation. Next, the findings were also presented to independent researchers; some additional data/quotes were then recommended to be included to support the interpretation.

The study has limitations. The present study was conducted at one institution, and therefore the findings are unable to be generalized to institutions. Meanwhile, the participants were from the bachelor’s program, while majority of nursing students in the country were in the diploma program. The Bachelor’s programme is aimed to train professionals with a high level of critical thinking and responsible for decision making [64]. In contrast, the diploma programme is aimed to produce technical individuals for support service [65]. Therefore, bachelor students may benefit from more cognitive-oriented learning compared to diploma students who rely more on skills-oriented learning [8] as online learning primarily caters to cognitive purpose [49–51]; thus, the acceptability of online learning in diploma nursing students is unable to be determined. More qualitative study is recommended to replicate this study in other nursing students’ populations, such as diploma students, and other institutions including public and private. Broader social representation of the participants should be considered, such as having a significant number of participants from significant ethnicities in Malaysia (i.e., Malay, Chinese, Indian, and other Bumiputera), as well as from a variety of economic (B40, M40, and T20), and geographical background (remote, rural and urban).

Nevertheless, the finding of this study is considered valuable among nursing students. Currently, the practice framework is merely a concept; thus, the efficacy of the practice framework in actual teaching and learning activity is unknown. It is advised that an experimental study be conducted in the future. However, as online learning is expected to become a norm in the future and many international institutions offer full online degree courses [66], the practice framework is believed to remain relevant.

In health, learners are not limited to student or junior health practitioners but also include clients who are patients and their family members. Client education, individually or in the group, as in community health promotion, is beneficial in elevating health quality [67]. The practice of client education utilizes various mediums such as written materials (e.g., leaflets), the internet, and mobile phone apps; however, lacking in utilizing standard instructional design or based on robust educational theory [67, 68, 69]. Most client education uses traditional lectures even though there is a trend of those education shifted from conventional clinical attendance to online [68, 69]. Implementing instructional design was found to have a desirable outcome for healthcare learners [70], which can be translated to client use. Client education may benefit from proper instructional design. Therefore, this framework can also be a valuable instructional design for client education.

## Conclusion

Online learning has become a mainstream medium for teaching and learning delivery. Nursing and other health sciences education should prepare to embrace such practice, and the COVID-19 situation has cemented the transition rapidly. Online learning can be made meaningful and interactive by structuring the activities with various didactic and active learning approaches. Students’ participation is emphasized and adapted to the online learning needs. The activities should foster students’ development in other areas beyond just education, such as social life and university experience. The health and wellbeing factors are given attention for online learning by incorporating healthy habits for computer use during teaching and learning. Good educators’ quality and evidence-based teaching and learning approaches underpin the success of online learning.

## Data Availability

The datasets generated during and/or analysed during the current study are not publicly available due to ethics/consent requirement but are available from the corresponding author on reasonable request.
